# Supplementary material for: Force-FAK signaling coupling at individual focal adhesions coordinates mechanosensing and microtissue repair
Source: Nat Commun. 2021 Apr 21;12:2359. doi: 10.1038/s41467-021-22602-5 (PMC8060400; doi:10.1038/s41467-021-22602-5)
Supplement: Supplementary file 1 — Supplementary Information [file 41467_2021_22602_MOESM1_ESM.pdf]

Supplementary Materials

Force-FAK Signaling Coupling at Individual Focal Adhesions Coordinates Mechanosensing and Microtissue Repair

Dennis W. Zhou[1,2†], Marc A. Fernández-Yagüe[2,3†], Elijah N. Holland[2,4], Andrés F. García[3], Nicolas S. Castro[1], Eric B. O'Neill[2,3], Jeroen Eyckmans[5,6], Christopher S. Chen[5,6], Jianping Fu[7], David D. Schlaepfer[8], Andrés J. García[2,3*]

[1]Coulter Department of Biomedical Engineering, Georgia Institute of Technology, Atlanta, GA 30332, USA
[2]Petit Institute for Bioengineering and Bioscience, Georgia Institute of Technology, Atlanta, GA 30332, USA
[3]Woodruff School of Mechanical Engineering, Georgia Institute of Technology, Atlanta, GA 30332, USA
[4]School of Chemical and Biomolecular Engineering, Georgia Institute of Technology, Atlanta, GA 30332, USA
[5]Department of Biomedical Engineering, Boston University, Boston, MA 02215, USA
[6]Wyss Institute for Biologically Inspired Engineering, Harvard University, Boston, MA 02115, USA
[7]Department of Mechanical Engineering, Department of Biomedical Engineering, Department of Cell and Developmental Biology, University of Michigan, Ann Arbor, MI 48109, USA
[8]Moores Cancer Center, Department of Obstetrics, Gynecology, and Reproductive Sciences, University of California, San Diego, La Jolla, CA 92093, USA

[†] These authors contributed equally to this work
[*]Corresponding author, andres.garcia@me.gatech.edu

This PDF file includes:

Supplementary Table 1
Supplementary Figures 1 - 14
Supplementary Movies 1-3

Supplementary Table 1. Parameters used in kinetic model.

symbol	parameter	units	value
T	unstretched talin	#	variable
T_{st}	stretched talin	#	variable
T_{total}	total talin	#	1000
F	free FAK	#	variable
FT_{st}	FAK-stretched talin complex	#	variable
pFT_{st}	phosphorylated FAK-stretched talin complex	#	variable
F_{total}	total FAK	#	1000
f	force applied to talin	pN	variable
α	force coupling factor	pN^{-1}	0 – 0.10
k_{1o}	basal forward rate for talin stretching	s^{-1}	1
k_1	forward rate for talin stretching	s^{-1}	variable
k_2	reverse rate for talin stretching	s^{-1}	10
k_3	forward binding rate for FAK-talin complex	$\#^{-1}\ s^{-1}$	0.001 – 0.1
k_4	reverse binding rate for FAK-talin complex	s^{-1}	10
k_5	FAK phosphorylation rate	s	0.03 - 3
k_6	FAK de-phosphorylation rate	s^{-1}	10

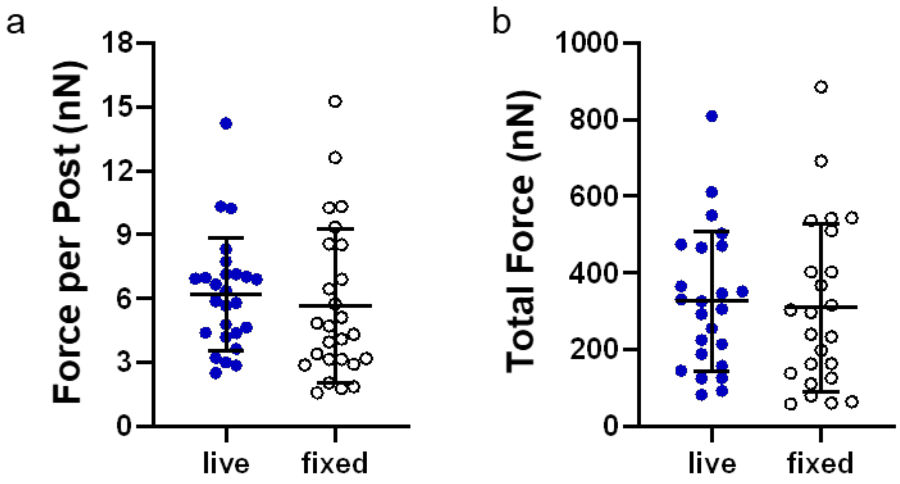

Supplementary Fig. 1. Fixation protocol maintains cell forces on mPADs. **a**, Force per post (mean ± SD, n=26 FAs for each group). Unpaired two-sided t-test P=0.5429. **b,** Total Force (mean ± SD, n=24 FAs for each group). Unpaired two-sided t-test P=0.7875.

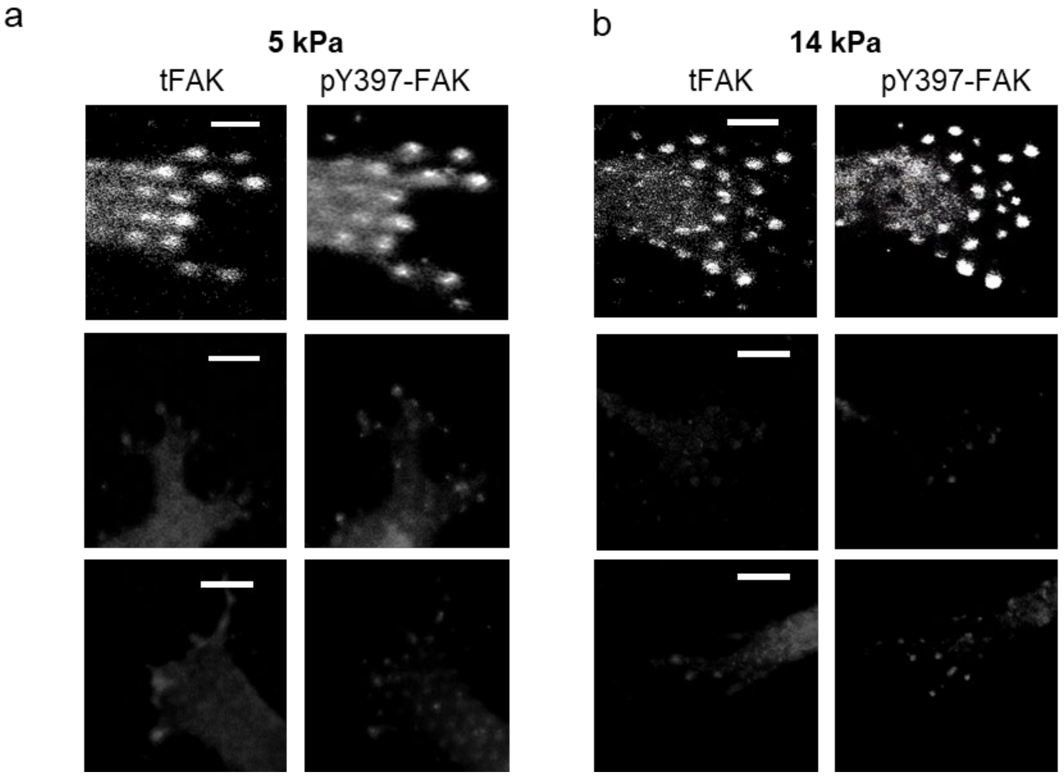

Supplementary Fig. 2. Immunostaining of tFAK and pY397-FAK in control cells and cells treated with Y-27632 or PF-228 for cells cultured on (**a**) 5 kPa mPADs and (**b**) 14 kPa mPADs. Scale bar 10 μm.

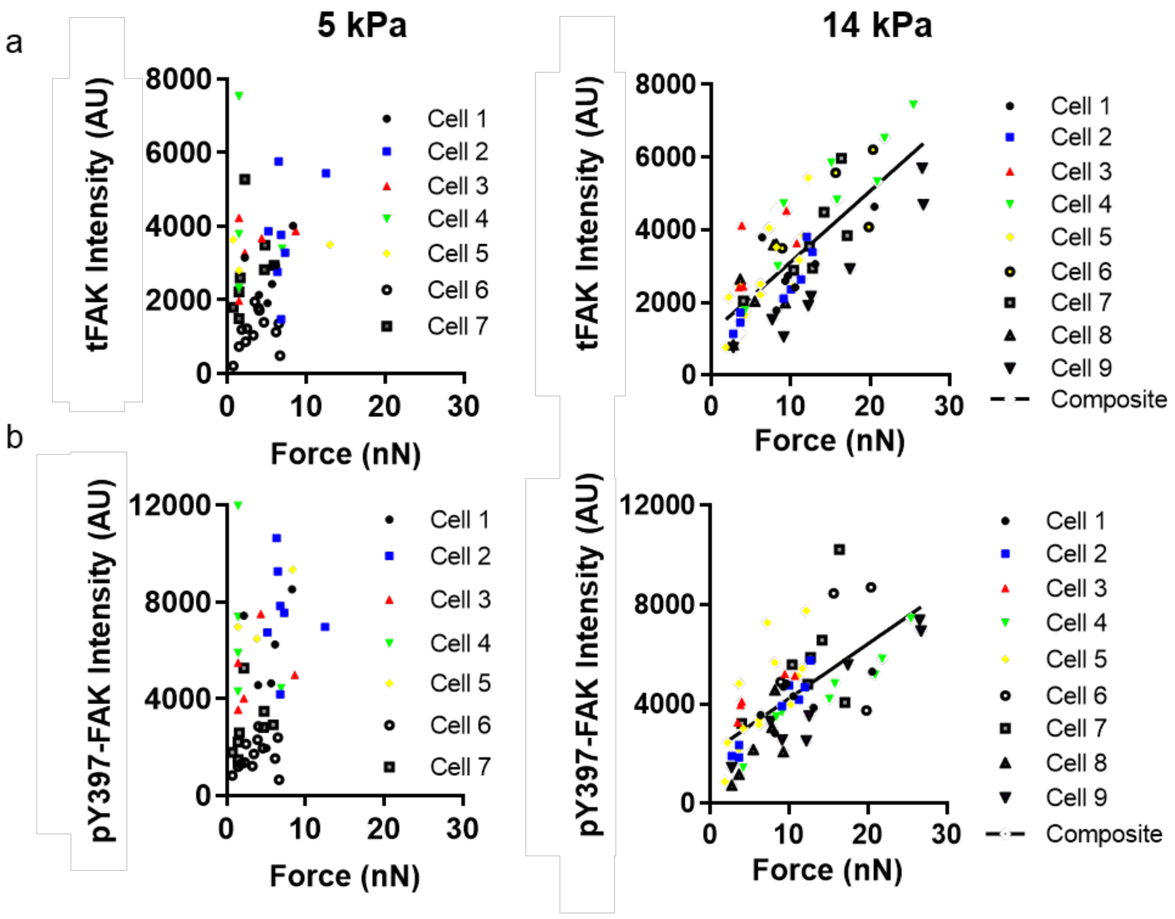

Supplementary Fig. 3. Linear relationship between traction force and tFAK and traction force and pY397-FAK at individual FAs for multiple cells. **a**, tFAK intensity as a function of force at individual FAs from multiple cells on 5 or 14 kPa mPADs. Linear regression: 5 kPa P=0.1230, n=50 FAs; 14 kPa P<0.0001, n=66 FAs, tFAK intensity = 194.6*force + 1178. **b**, pY397-FAK intensity as a function of force at individual FAs from multiple cells on 5 or 14 kPa mPADs. Linear regression: 5 kPa P=0.1072, n=50 FAs; 14 kPa P<0.0001, n=66 FAs, pY397-FAK intensity = 219.7*force + 2027.

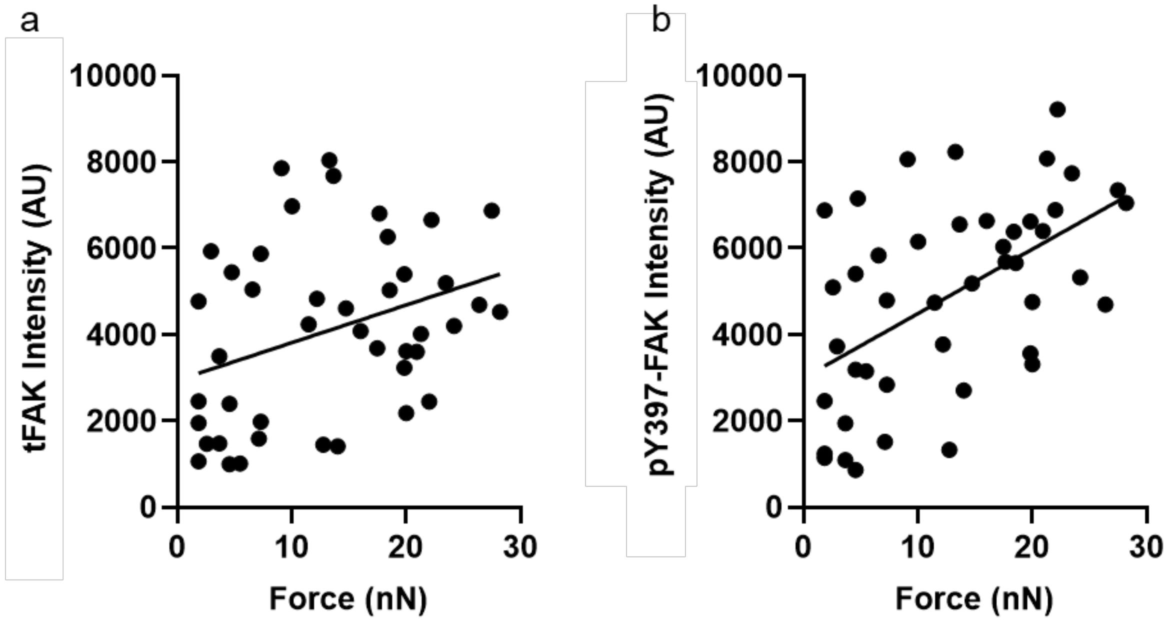

Supplementary Fig. 4. Force-FAK signaling linear coupling at individual FAs for human mesenchymal stem cells on 14 kPa mPADs. **a**, Force-tFAK relationship. Linear regression: P=0.0211, n=44 FAs, tFAK intensity = 87.17*force + 2940. **b**, Force-pY397-FAK relationship. Linear regression: P=0.0002, n=44 FAs, pY397-FAK intensity = 149.0*force + 2996.

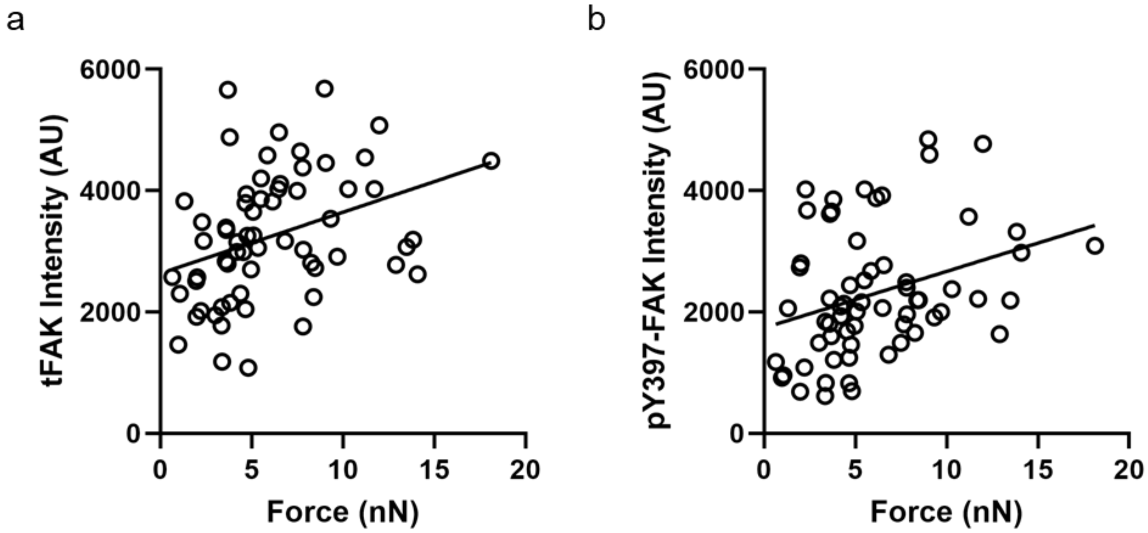

Supplementary Fig. 5. Force-FAK signaling linear coupling at individual FAs for fibroblasts on 25 kPa mPADs. **a**, Force-tFAK relationship. Linear regression: P=0.0043, n=63 FAs, tFAK intensity = 100.8*force + 2637. **b**, Force-pY397-FAK relationship. Linear regression: P=0.0094, n=63 FAs, pY397-FAK intensity = 93.2*force + 1739.

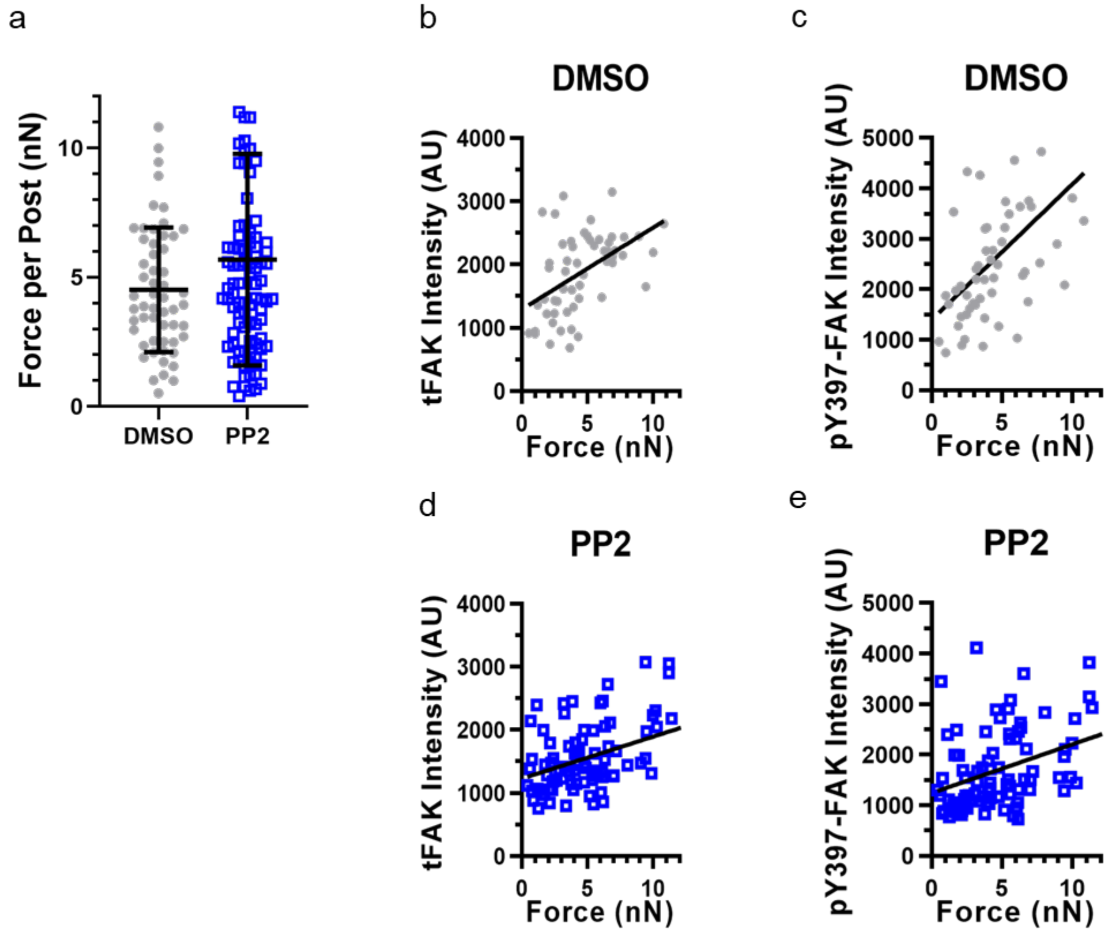

Supplementary Fig. 6. Effect of inhibition of Src-family kinases on force-FAK signaling coupling. **a**, PP2 treatment does not alter traction force (mean ± SD) at individual FAs compared to controls. Mann-Whitney test P=0.2541, n=55 and 95 FAs for DMSO and PP2, respectively. **b**, Force vs tFAK intensity at individual FAs on 14 kPa for DMSO control. Linear regression: P<0.0001, n=55 FAs, tFAK intensity = 129.3*force + 1294. **c**, Force vs pY397-FAK intensity at individual FAs on 14 kPa for DMSO control. Linear regression: P<0.0001, n=55 FAs, pY397-FAK intensity = 268.8*force + 1396. **d**, Force vs tFAK intensity at individual FAs on 14 kPa for PP2-treated cells. Linear regression: P<0.0001, n=95 FAs, tFAK intensity = 66.1*force + 1230. **e**, Force vs pY397-FAK intensity at individual FAs on 14 kPa for PP2-treated cells. Linear regression: P<0.0001, n=95 FAs, pY397-FAK intensity = 96.2*force + 1244.

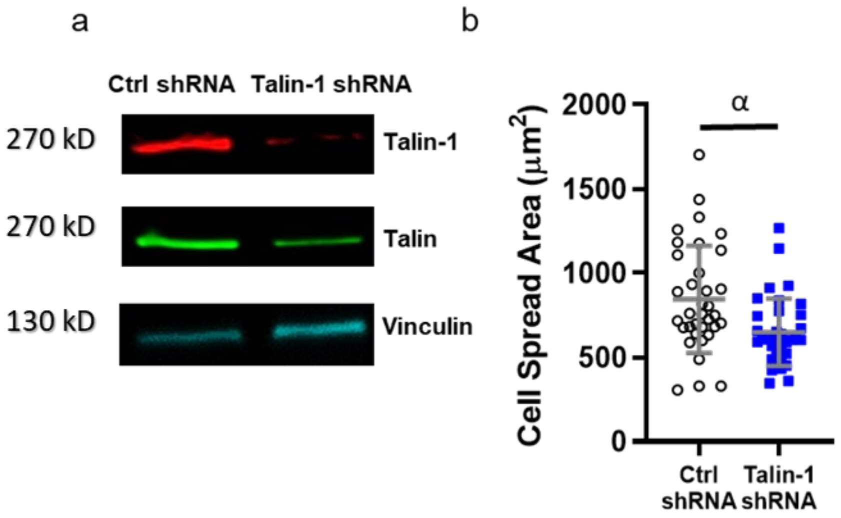

Supplementary Fig. 7. Talin-1 promotes cell spreading and traction force generation. **a,** Western blots confirming efficient talin-1 depletion in talin-1 shRNA-treated cells compared to ctrl shRNA-treated cells. Vinculin was used as a loading control. **b,** Effects of talin-1 depletion on cell spreading (mean ± SD). Two-sided Mann-Whitney test α: P=0.0014, n=35 cells.

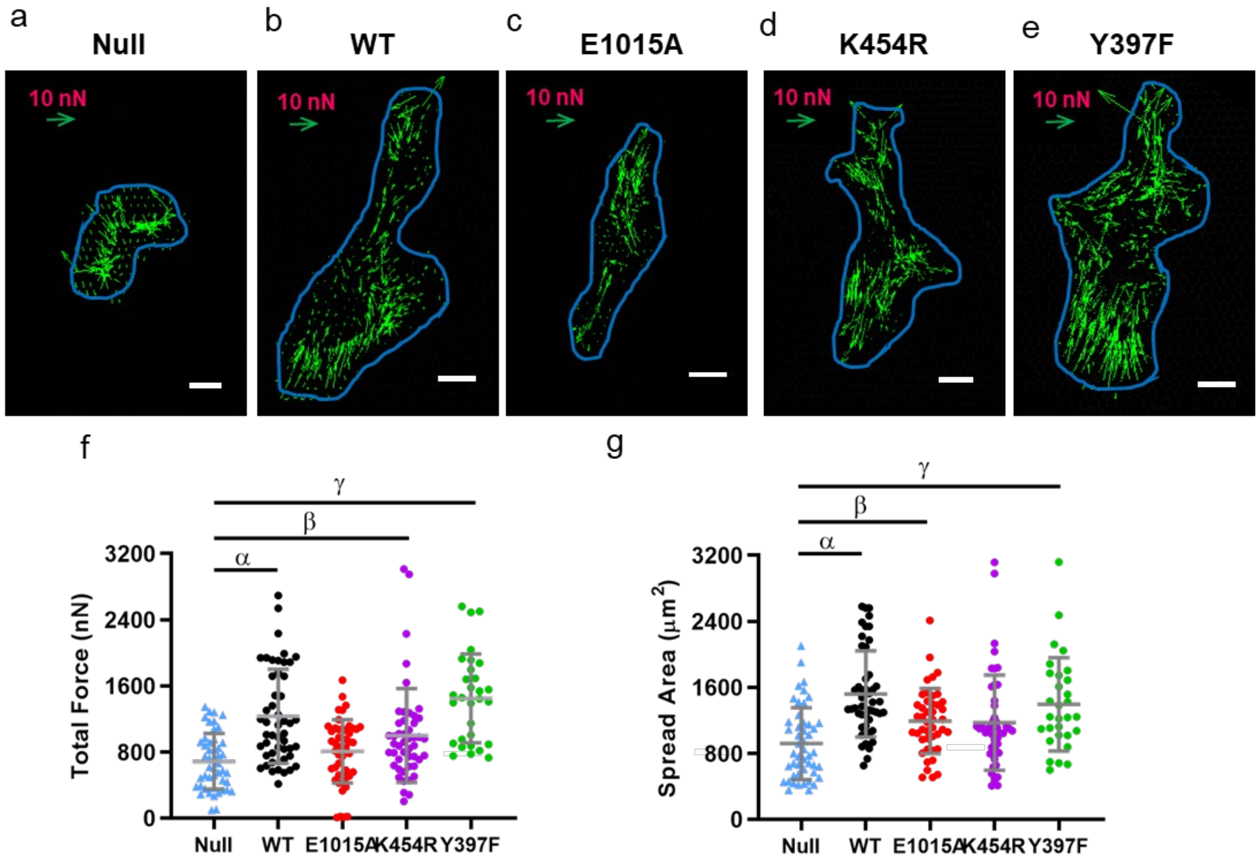

Supplementary Fig. 8. Traction force and cell spreading for FAK-null cells expressing FAK constructs on 14 kPa mPADs. **a-e**, FAK construct-expressing cells generate traction forces on mPADs (force vectors [green] and cell outline [blue]). Scale bar 20 μm. **f**, Total traction force (mean ± SD). Two-sided Kruskal-Wallis test P<0.0001, α: P<0.0001, β: P=0.0401, γ: P<0.0001, n=48, 48, 44, 47, 30 cells for each condition, respectively. **g**, Spread area (mean ± SD). Two-sided Kruskal-Wallis test P<0.0001, α: P<0.0001, β: P=0.0346, γ: P=0.0009, n = 49, 49, 44, 47, 30 cells for each condition, respectively.

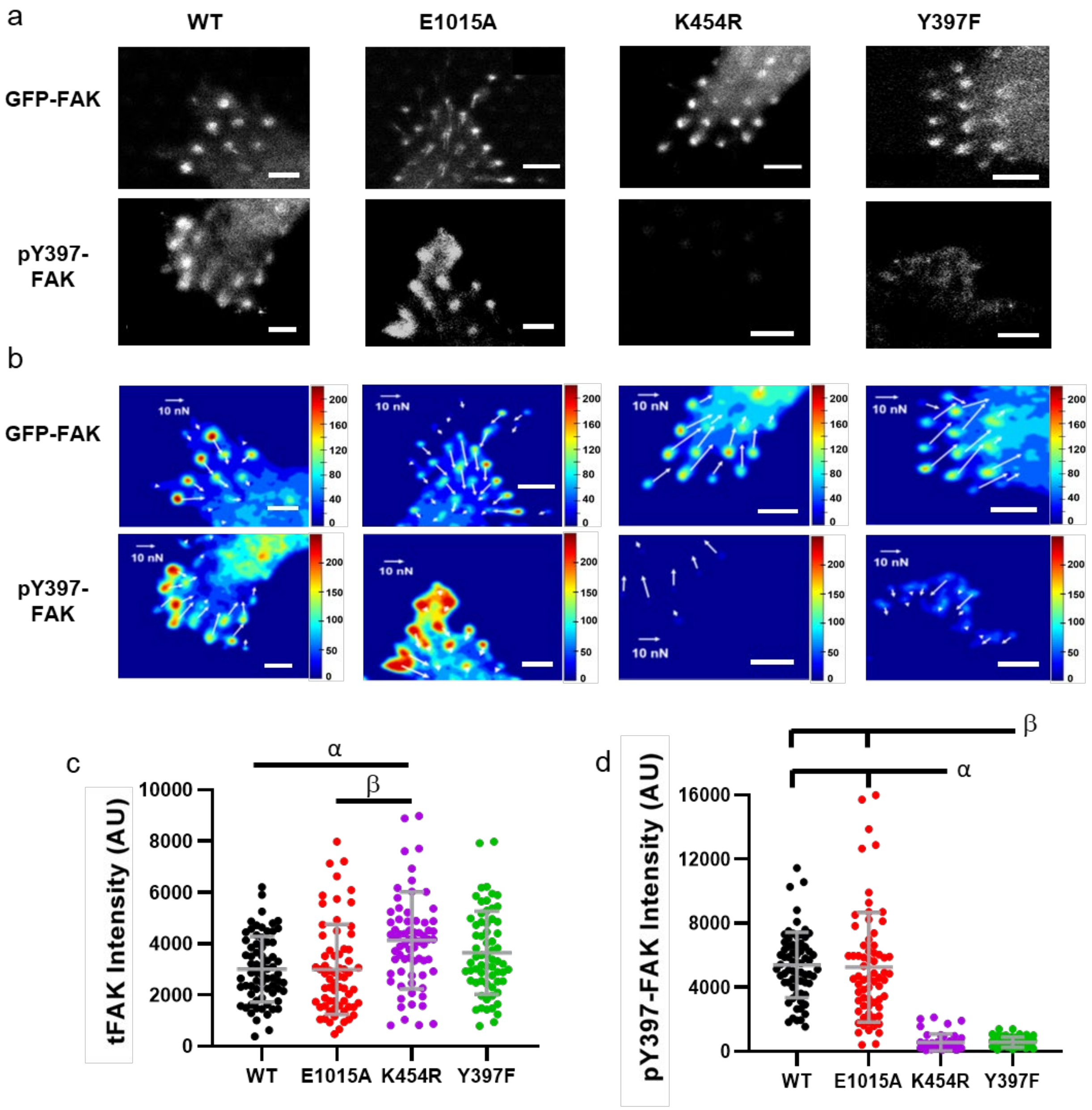

Supplementary Fig. 9. Immunostaining and heatmaps for FAK-null cells expressing FAK constructs. **a,** GFP-FAK and pY397-FAK images from FAK mutant-expressing cells. Scale bar 5 μm. **b,** Heatmaps (arbitrary units) of GFP-FAK and pY397-FAK staining on 14 kPa mPADs. White arrows indicate force magnitudes and directions. Scale bar 5 μm. **c,** tFAK intensity (mean ± SD) at individual FAs. ANOVA $P<0.0001$, α: $P=0.0004$, β: $P=0.0005$, n=70, 66, 70, 65 FAs for each condition, respectively. **d,** pY397-FAK intensity (mean ± SD) at individual FAs. ANOVA with Welch's correction $P<0.0001$, α: $P<0.0001$, β: $P<0.0001$, n=69, 68, 47, 60 FAs for each condition, respectively.

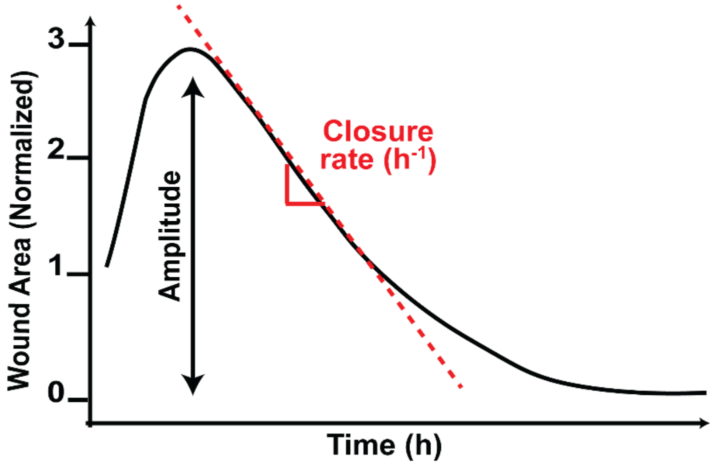

Supplementary Fig. 10. Schematic of log-normal curve fit for microtissue wound healing profile showing amplitude and wound closure rate parameters.

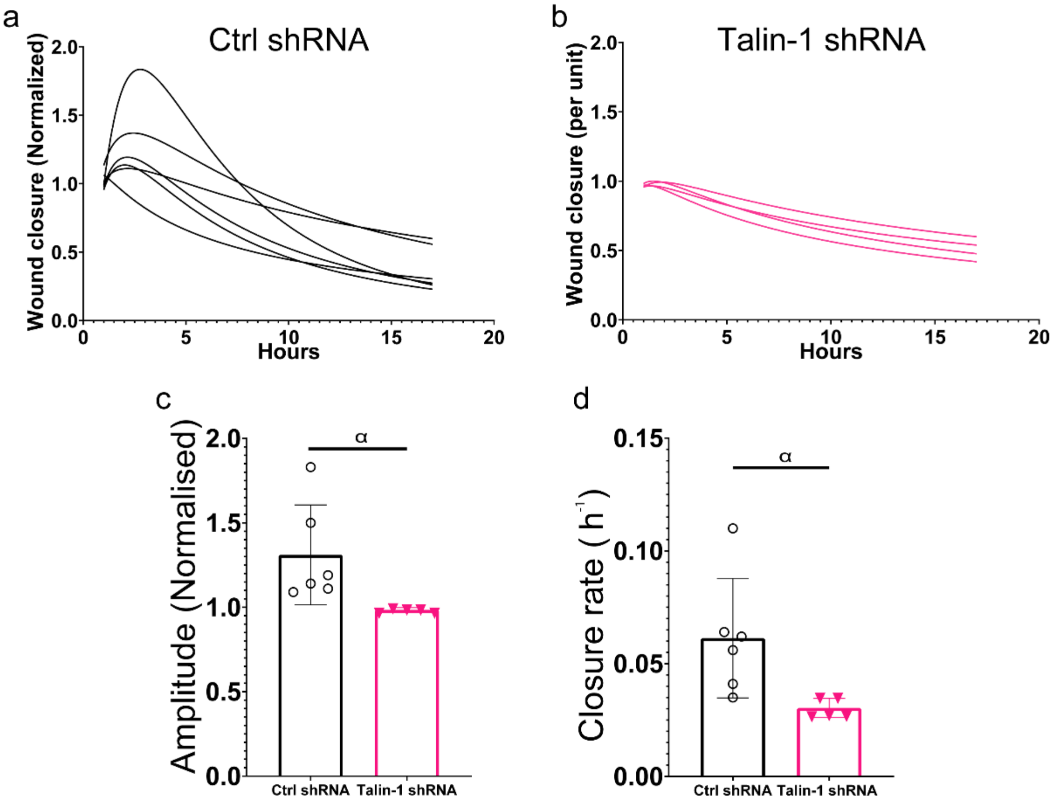

Supplementary Fig. 11. Wound closure for microtissues containing control shRNA-treated or talin-1 shRNA-treated fibroblasts. **a**,**b** Log-normal curve fit for wound area normalized to initial area as a function of time for individual microtissues seeded with (**a**) control shRNA-treated or (**b**) talin-1 shRNA-treated fibroblasts, n=6 independent microtissues from 2 independent experimental runs. **c**, Amplitude (mean ± SD) of normalized wound area profile. Two-sided Mann-Whitney test α: P=0.0043, n=6 independent microtissues from 2 independent experimental runs. **d**, Wound closure rate (mean ± SD) of normalized wound area profile. Two-sided Mann-Whitney test α: P=0.0087, n=6 independent microtissues from 2 independent experimental runs.

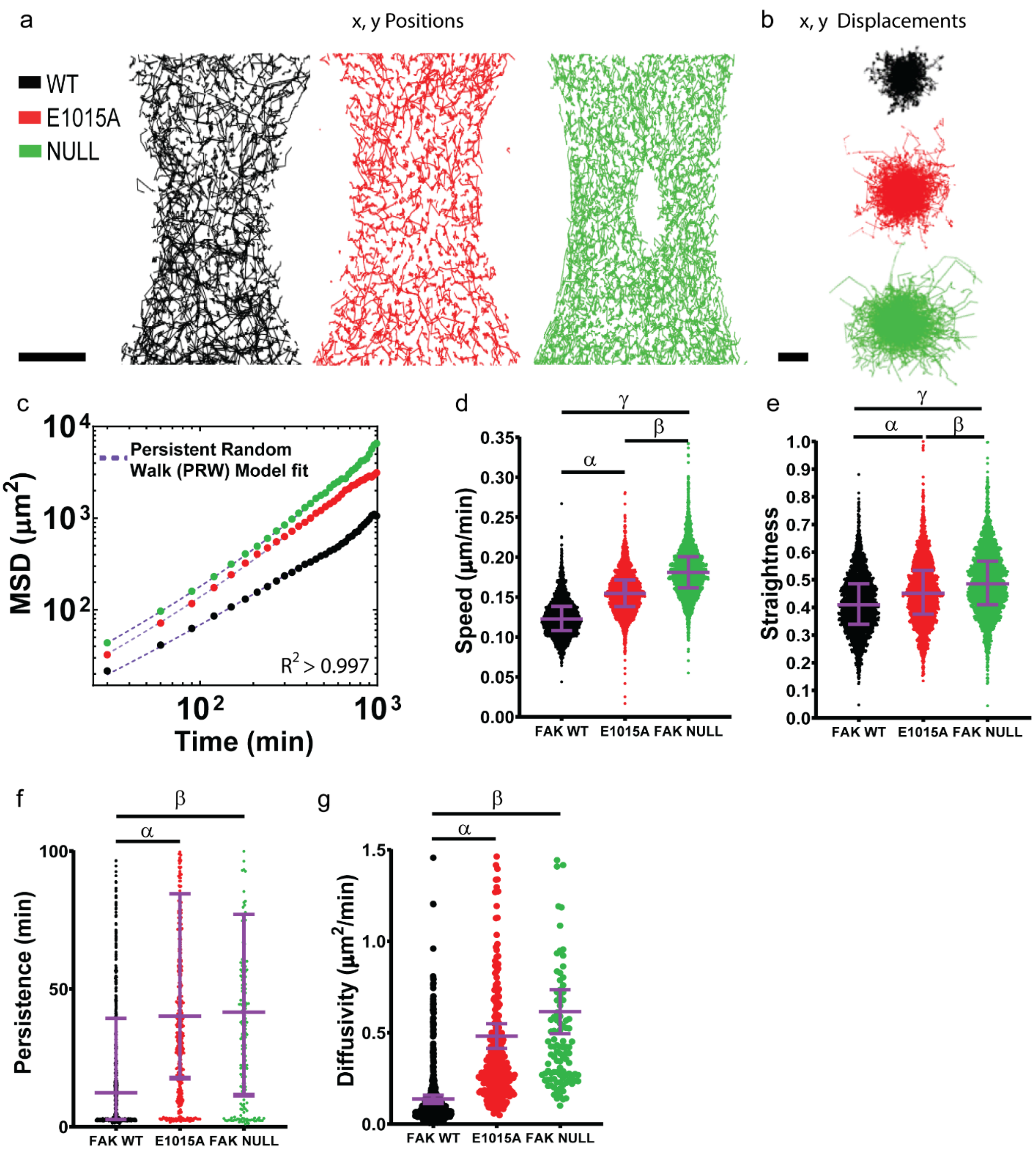

Supplementary Fig. 12. Cell migration during microtissue repair for FAK-null cells expressing FAK constructs. **a**, Motility vectors for individual cells on microtissues. Scale bar 250 μm. **b**, Cell tracks. Scale bar 50 μm. **c**, Mean square displacement (MSD) over time and Persistent Random Walk model fit (n=2391 cells from 5 microtissues for WT and 6 microtissues for null and E1015A from 2 independent experimental runs). **d**, Cell speed (median ± IQR). Nested ANOVA $P<0.0001$, α: $P=0.0003$, β: $P=0.0025$, γ: $P<0.0001$, n=2391 cells from 5 microtissues for WT and 6 microtissues for null and E1015A from 2 independent experimental runs). **e**, Straightness index (median ± IQR). Nested ANOVA $P<0.0001$, α: $P=0.0015$, β: $P=0.0051$, γ: $P<0.0001$, n=2353 cells

from 5 microtissues for WT and 6 microtissues for null and E1015A from 2 independent experimental runs. **f**, Persistence (median ± IQR). Nested Kruskal-Wallis test P=0.0002, α: P=0.0202, β: P=0.0009, n=560 cells from n=5 microtissues for WT and 6 microtissues for null and E1015A from 2 independent experimental runs. **g**, Diffusivity (median ± IQR). Nested Kruskal-Wallis test P<0.0001 α: P=0.0284, β: P=0.0006, n=757 cells from 5 microtissues for WT and 6 microtissues for null and E1015A from 2 independent experimental runs.

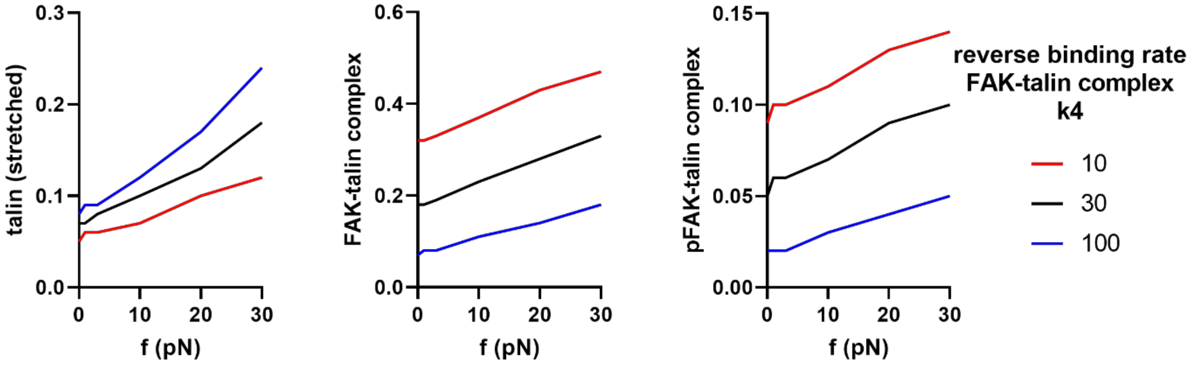

Supplementary Fig. 13. Solutions for the relative number of stretched talin, FAK-talin complexes, and phosphorylated FAK-talin complexes as a function of applied force (f) for different values of the reverse binding rate constant for FAK-talin complex (k_4).

a Western blots for Figure 4

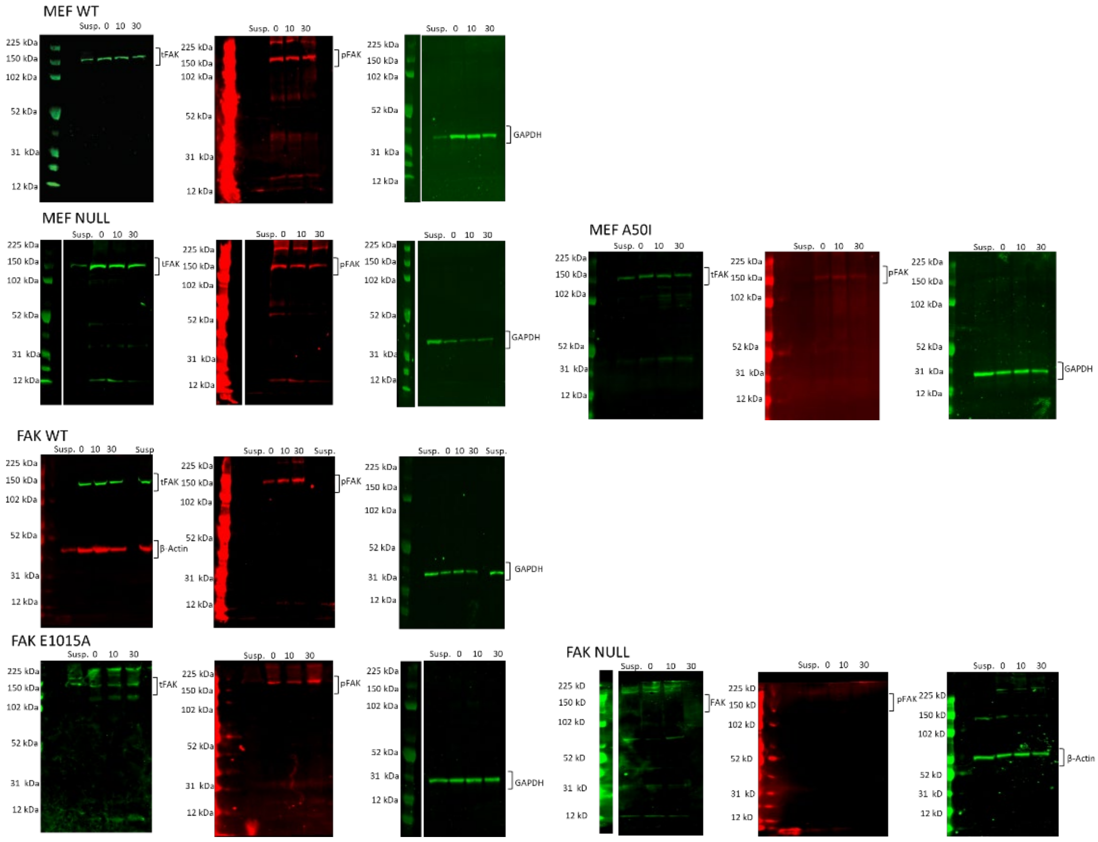

b Western blots for Supplementary Figure 7

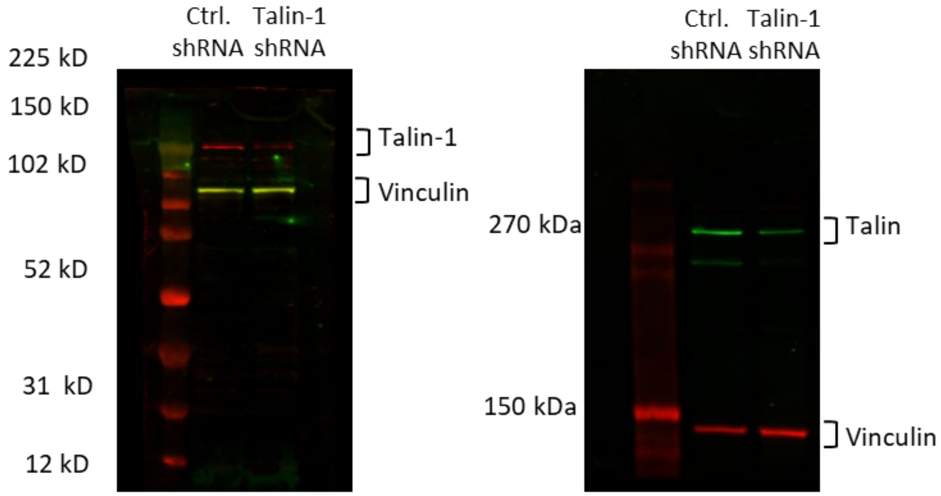

Supplementary Fig. 14. Uncropped Western blots for (a) Figure 4 and (b) Supplementary Figure 7.
