# Supplementary material for: Force-FAK signaling coupling at individual focal adhesions coordinates mechanosensing and microtissue repair
Source: Nat Commun. 2021 Apr 21;12:2359. doi: 10.1038/s41467-021-22602-5 (PMC8060400; doi:10.1038/s41467-021-22602-5)
Supplement: Supplementary file 3 — Description of Additional Supplementary Files [file 41467_2021_22602_MOESM3_ESM.docx]

**Description of Additional Supplementary Files**

File Name: **Supplementary Movie 1.**

Description: Microtissue containing WT FAK-expressing cells following wounding.

File Name: **Supplementary Movie 2.**

Description: Microtissue containing E1015A FAK-expressing cells following wounding.

File Name: **Supplementary Movie 3.**

Description: Microtissue containing FAK-null cells following wounding.
