# Supplementary material for: Force-FAK signaling coupling at individual focal adhesions coordinates mechanosensing and microtissue repair
Source: Nat Commun. 2021 Apr 21;12:2359. doi: 10.1038/s41467-021-22602-5 (PMC8060400; doi:10.1038/s41467-021-22602-5)
Supplement: Supplementary file 7 — Reporting Summary [file 41467_2021_22602_MOESM7_ESM.pdf]

nature research

Corresponding author(s): Andrés J. García

Reporting Summary

Nature Research wishes to improve the reproducibility of the work that we publish. This form provides structure for consistency and transparency in reporting. For further information on Nature Research policies, see Authors & Referees and the Editorial Policy Checklist.

Statistical parameters

When statistical analyses are reported, confirm that the following items are present in the relevant location (e.g. figure legend, table legend, main text, or Methods section).

n/a	Confirmed	
☐	☒	The exact sample size (n) for each experimental group/condition, given as a discrete number and unit of measurement
☐	☒	An indication of whether measurements were taken from distinct samples or whether the same sample was measured repeatedly
☐	☒	The statistical test(s) used AND whether they are one- or two-sided<br>*Only common tests should be described solely by name; describe more complex techniques in the Methods section.*
☒	☐	A description of all covariates tested
☐	☒	A description of any assumptions or corrections, such as tests of normality and adjustment for multiple comparisons
☐	☒	A full description of the statistics including central tendency (e.g. means) or other basic estimates (e.g. regression coefficient) AND variation (e.g. standard deviation) or associated estimates of uncertainty (e.g. confidence intervals)
☐	☒	For null hypothesis testing, the test statistic (e.g. F, t, r) with confidence intervals, effect sizes, degrees of freedom and P value noted<br>*Give P values as exact values whenever suitable.*
☒	☐	For Bayesian analysis, information on the choice of priors and Markov chain Monte Carlo settings
☒	☐	For hierarchical and complex designs, identification of the appropriate level for tests and full reporting of outcomes
☒	☐	Estimates of effect sizes (e.g. Cohen's d, Pearson's r), indicating how they were calculated
☐	☒	Clearly defined error bars<br>*State explicitly what error bars represent (e.g. SD, SE, CI)*

Our web collection on statistics for biologists may be useful.

Software and code

Policy information about availability of computer code

Data collection	Nikon C2 Confocal NIS Elements Software 4.11, Image Studio 5.0 (LI-COR Biosciences)
Data analysis	Graphpad Prism 8.1.1, NIS-Elements 4.11, ImageJ 1.53c, Custom MATLAB (R2019b) code for analyzing mPADs displacement (from Jianping Fu's group, available upon request), Image Studio 5.0 (LI-COR Biosciences) for analyzing WBs, COMSOL 5.4 for force calculation by magnetic beads.

For manuscripts utilizing custom algorithms or software that are central to the research but not yet described in published literature, software must be made available to editors/reviewers upon request. We strongly encourage code deposition in a community repository (e.g. GitHub). See the Nature Research guidelines for submitting code & software for further information.

Data

Policy information about availability of data

All manuscripts must include a data availability statement. This statement should provide the following information, where applicable:

- Accession codes, unique identifiers, or web links for publicly available datasets
- A list of figures that have associated raw data
- A description of any restrictions on data availability

The data that support the plots within this paper are available from the corresponding author upon request.

Field-specific reporting

Please select the best fit for your research. If you are not sure, read the appropriate sections before making your selection.

- [x] Life sciences
- [ ] Behavioural & social sciences

For a reference copy of the document with all sections, see nature.com/authors/policies/ReportingSummary-flat.pdf

Life sciences

Study design

All studies must disclose on these points even when the disclosure is negative.

Sample size	All experiments were conducted with at least two separate mPAD samples per condition. Sample sizes for number of cells and FAs analyzed were determined based on our previous experience and similar studies of other groups.
Data exclusions	Cells that were contacting other cells were not included in our analyses.
Replication	All attempts at replication were successful.
Randomization	Samples were randomly allocated to control and experimental groups.
Blinding	Investigators were not blinded to group allocation, as no animal/human studies were conducted in this manuscript.

Materials & experimental systems

Policy information about availability of materials

n/a	Involved in the study
[ ]	[x] Unique materials
[ ]	[x] Antibodies
[ ]	[x] Eukaryotic cell lines
[x]	[ ] Research animals
[x]	[ ] Human research participants

Unique materials

Obtaining unique materials	mPADs are available upon request from Jianping Fu or Andres Garcia. Molds for microtissues are available upon request from Christopher Chen.

Antibodies

Antibodies used	FAK (EMD Millipore, Clone 4.47, 05-537, 1:200, IF; 1:500, WB), pY397-FAK (Abcam, ab39967 for Vinculin MEFs – 1:300, ab81298 for hMSCs – 1:200, IF; Cell Signaling, 3283S,1:250,WB), beta-Actin (Cell Signaling, 8457S, D6A8, 1:100,WB), GAPDH (Abcam, ab8245, Clone 6C5 , 1:1000, WB), Talin-1 (Abcam, ab71333, 1 µg/mL, WB), Talin (Sigma, 8d4, 1:200, WB), Vinculin (Sigma, V284, 1:8000, WB), IRDye® CW 800 Goat anti-Rabbit IgG (H+L) (LICOR Biosciences, 925-32210, 1:7500, WB), IRDye® 680 RD Goat anti-Mouse IgG (H+L) (LICOR Biosciences, 926-68070, 1:7500, WB), AlexaFluor 405 Goat anti-Rabbit IgG (H+L) (Jackson Labs, 1:200), AlexaFluor 555 Goat anti-Mouse IgG (H+L) (Thermo Scientific,1:200), Mouse IgG1 Negative Control (1:200), clone Ci4 (EMD Millipore,1:250, IF), Phalloidin-iFluor 647 (abcam, ab176759,1:100), Alexa Fluor® 594 Goat Anti-Rabbit IgG Fc preadsorbed (abcam, ab150100, 1:200,IF), Goat Anti-Mouse IgG1 heavy chain (FITC) preadsorbed (abcam, ab98692,1:200,IF).<br><br>The antibody information (including species, application, and catalog number) has been provided in the methods. All antibodies have been validated by the companies from which they were purchased from. The subcellular localization of all the proteins analyzed in this work has been previously reported, and this information was used to further validate the specificity of antibodies.
Validation	Antibodies were validated for use in this system using positive and negative control samples using dilutions and conditions based on the manufacturer's recommendation or our previous experience. For instance, the pY397-FAK antibody was validated by staining cells with and without PF-228 (FAK inhibitor).

Eukaryotic cell lines

Policy information about cell lines

Cell line source(s)	Mouse Embryonic Fibroblasts (MEFs): Vinculin-null mouse embryonic fibroblasts (MEFs) expressing vinculin constructs were previously described (Dumbauld et al., PNAS 2013). Parental cells were provided by Eileen Adamson (Burnham Institute, La Jolla, CA). Subsequently, vinculin null MEFs were transduced with retrovirus to express the eGFP-tagged vinculin constructs.<br><br>FAK mutant MEFs were provided by David Schlaepfer (UCSD), where FAK-null MEFs were transduced with retrovirus to express GFP-FAK constructs.
Authentication	Reconstituted expression of eGFP-Vinculin and GFP-FAK constructs was validated using Western blotting and confocal imaging.
Mycoplasma contamination	WT vinculin expressing MEFs were tested in house for Mycoplasma contamination twice and found to be negative for contamination both times.
Commonly misidentified lines (See ICLAC register)	None of the cells used are listed in ICLAC.

Method-specific reporting

n/a	Involved in the study
☒	☐ ChIP-seq
☒	☐ Flow cytometry
☒	☐ Magnetic resonance imaging
